# Study of Defensive Methods and Mechanisms in Developmental, Emotional (Internalization), and Disruptive Behavior (Externalization) Disorders

**DOI:** 10.5539/gjhs.v6n7p109

**Published:** 2014-09-18

**Authors:** H. R. Jamilian, N. Zamani, M. Darvishi, M. R. Khansari

**Affiliations:** 1 Department of Psychiatry, Arak University of Medical Sciences, Arak, Iran; 2 Hamedan Sciences and Research Branch, Islamic Azad University, Graduate Campus, Hamedan, Iran

**Keywords:** defensive methods, defensive mechanisms, developmental, emotional, internalization, disruptive behavior, externalization

## Abstract

We need to find a way for adaptation with inherent unpleasantness of being human condition and conflicts that it caused, as we did not fail. Methods that we used for adaptation are named defense. This research have performed with the aim of study and compare defensive mechanisms and methods of Developmental, Emotional (Internalization), and Disruptive behavior (Externalization) disorders. Method, sample of this research included 390 family that are by available sampling method are selected. Tools of research were structured clinical interview of forth cognitive and statistical guide of psychopathic disorders for axis I and the way used for assess defensive mechanisms is defensive method 40 question’s questionnaires of Andrews (1993). The data are compared by statistical methods comparison of averages and one way variance analysis and HSD tests and results show that undeveloped defensive mechanisms in by developmental disorder family(25.2± 3.7) mean and standard deviation, it is most used mechanism and in disruptive behavior disorder family by (11.2 ±1.9) mean and standard deviation is used least mechanism and in developed mechanism of emotional disorder family by (7.8 ± 3.1) mean and standard deviation is most used mechanism and in developmental disorder family by (4.3 ±1.5) mean and standard deviation is least mechanism in neuroticism patient, social phobia affected emotional disorder family (15.6±2.6) and disruptive behavior disorder family have least mean and standard deviation(9.2±1.7) (p< 0.005). Recent research shows significant of study defensive mechanism in psychopathic family of disorder children that affecting on the way of life of persons and interpersonal and intrapersonal relations and method of solving problem in family of them in life, so defensive mechanisms require more attention.

## 1. Introduction

Innovation of psychoanalysis is second revolution in psychotherapist science (Shojaei Shefti, 2001) that is one of the effective and dynamic plans and suitable for today agitated society (Costa et al., 1995) and it is general term that refer to all models of mind which are about unconscious processes. Origin of all models of psycho gnosis (and some of them that are present don’t follow any sight of psycho gnosis) referred to Freud and psychoanalysis (Holmens, 1994).

Psychoanalysis system of Freud has great effect on theory and action in psychology and psychotherapist and our vision about nature of human and our perception of identity (Afzali et al., 2008). One basic assumption or hypothesis that is distinct it from psycho gnosis model is that, it is dynamic unconsciously and so it is source of motivation of our behavior, feelings and thoughts, not only thing that we are not aware about them (Solms, 2004) as said, psychoanalysis proceed with interpretation of speech and expression of patient, according to meanings, motivations and structure of thoughts (Spiegel, 1994) and main shafts of theory, accept unconscious mental processes, accept resistance and regression, confirm importance of Eros and invasion and Oedipus complex (Joseph, 1994) and it is used simple way of mental purification (catarzis) to remember thoughts in contemporaneous time in order to decrease increasingly nervous stresses (Gabbard, 1995).

Psycho gnosis models consider as start point of this design that we have intrinsic world which has great effect on how is thought, feeling and our behavior. Our intrinsic world is comprised of feeling, memories, beliefs and fantasies. A part of it is conscious, it means we know it and has access to it but most part of it is unconscious, it means that according to its definition we are uninformed and we don’t easily access to it (Baker, 1993).

Freud discusses three systems or level of consciousness: in conscious, semiconscious, unconscious (Green, 2003). Conscious mind act on level and it is related to thing that we are aware about them or thinking about it, under it there is semiconscious mind that we can hold all memories, feelings and beliefs which can easily access to it but there are not in conscious part of our mind, there is different depth of semiconscious of our mind; access to some material of semiconscious of our mind is so easy. Unconscious part is under semiconscious part and it is source of intellectual subjects that are so stressful for allow enter to conscious part which is including rugged and sexual impulsive, defenses and some memories and feeling (Holmens, 2003).unconscious mind follow from a collection rules of conscious mind. He argued that existence of unconscious mind is under control of primary processes, whereas existence of conscious mind is control by secondary processes (Holmens, 1994; Spiegel, 1994). His mean was that conscious mind base on logic regulations are organized, it associated with reality, it can defer willing and understand concepts of time and difference among objects and persons. In contrast primary processes or unconscious part of mind are not about reality (Brouer & Freud, 1994).

We need to find a way for adaptation by inherent unpleasantness of being human condition and conflicts that it caused, as we did not fail. Methods that we used for adaptation are named defense. We can properly use defenses methods consciously and intentionally, but psycho gnosis model especially interested in defenses that we use it unconsciously. Frued found that this is a way for keep every willing unconsciously or thought that is threatening and it can source of anxiety. He knows aim of psychoanalysis is make conscious unconscious part through cancel regression (Fest & Fest, 2002). In order to manage adaptation challenges with unavoidable pleasantness of being human condition, we are formed our defenses at the beginning of our life. We need to our defenses so we can live in the world; therefore aim of psycho gnosis is not cancel them. This is not wise but also impossible. Nonetheless we need defenses that are flexible adequately for provide this possibility that according special situation we locate in it show reaction, as in all situations behave in a way that everything is the same. The defenses that are applied incompliance, they are not useful because decrease options for show how reaction we have and there is this probability that we even make a big problem for ourselves, because we have show improperly reaction. If a defense has applied incompliance, how it can prevent actual understanding (Freud, 1905/1953a).

Defenses are a part of our character and it is an important index for personal ways of our relation with the world. Thought of psycho gnosis interested in how we can apply every experience or feeling defensibly. Frued argued that the most important instinct is our sexual impulsive and more psychological defenses because of keep away from instinctive request for warning of consciousness part of our mind (Goodman et al., 1989). When one is beset by three sides of different hostile forces and predictability show reaction: become anxious. Then, it forced for defense from him against this anxious, it is used repression and other defensive mechanisms (Freud, 1905/1953b).

Frued for the first time create defensive mechanism concept in 1926 (Freud, 1905/1953b). And then his daughter Ana Frued modified and organized this concept. Although defensive mechanisms are normal and all people used them, but if it used extremely, it caused obsessive and repeated behavior and neuroticism behavior. Because for creating and defensive mechanisms must we use mental power, as we are more defensive, so we need less mental power for satisfying action of character. Of course this is actually oneself duty for creating defensive mechanisms, because it must avoid directs conflict with intrinsic request and defense from him against anxious along with them (Freud, 1905/1953a).

In psychoanalysis system every psychopathic disorder accompanied with specific unadoptable defensive mechanisms (Cramer, 2000; Andrews et al., 1993; Bond & Perry, 2004) and defenses have an important role in mental health of people, besides identifying defenses of different disorders for help recognize discrimination have acceptable application (Cramer, 2000). Freud declared personal defensible method; it means frequency use of defensive mechanisms in comparison with others is main variable for recognize personality, pathology and level of compatibility. Assumption that base researching findings has highly confirmed (Besharat, 2007a).

Main defensive mechanisms that Freud is specified include: repression, operation-de, compartmentalization, inverse action, displacement, stabilization, regression, projection, introjections and sublimation (Freud, 1905/1960). According to psychoanalysis process, people in opposed to stress used specified defensive methods, these methods based on level of maturity divided in to four groups: immature (underdeveloped), neuroticism, narcissistic and matured (developed) groups (Vaillant, 1992).

Each of these methods includes especial defensive mechanisms, and in people who affected to psychopathic disorders, undeveloped and unadapted defensive method of nonclinical population infinitely defensive method is more developed (Cramer, 2000). Hence things that perform in most researches related to psychotic defenses, first study of defensive method and second predominant defensive mechanisms that are people used them.

Therefore, the aim of present research is study and comparison defensive methods and mechanisms of people who affected by Developmental, Emotional (Internalization), and Disruptive behavior (Externalization) disorders.

## 2. Method

Design of present research is descriptive- analytic that its aim is state relation between phenomena and enhances existence collection knowledge about role of defensive mechanisms.

Statistical universe of this research include all people who according to reviewed context fourth edition of cognitive and statistical booklet of psychopathic disorders in American psychotherapist institute comprise of cognitive criteria of developmental, emotional (Internalization), and disruptive behavior (Externalization) disorders. sample of research was include 360 family of children disorder affected by psychopathic disorders that in 1392 have referred to private clinic centers and psychopathic and special school of Hamedan, among these family of children 390 family affected by psychopathic childhood and teenager disorders have criteria for enter in this research. These criteria include: 1) involved cognitive criteria of psychopathic disorders according to revision fourth edition of cognitive and statistical booklet of psychopathic disorders in American psychotherapist Institute and structured clinical interviewed of forth cognitive and statistical guide of psychopathic disorders for axis1 and psychotherapist diagnostic; 2) at least they have diploma degree; 3) range of their age between 19-43.

### 2.1 Omission Criteria Include

1) Classification psychopathic disorders axis II and III;
2) Psychotic patient;
3) Patients who hospitalized at psychical hospital.

Sampling has done according to available sampling method. Thus person who have needed selected criteria and have criteria for participate in this research underway about research and if they had satisfied, they selected as sample of research and complete questionnaire.

## 2.2 Tools of Research Included

1) Structured clinical interview of forth cognitive and statistical guide of psychopathic disorders for axis I;
2) Questionnaire of defensive methods.

SCID is a structured clinical interview for assessing different kinds of disorders axis I and II. This diagnostic interview for the first time in 90decade for assess according to reviewed context of third edition of cognitive and statistical booklet of psychopathic disorders in American psychotherapist institute is developed and its present type according to diagnostic assessment based on fourth edition of cognitive and statistical booklet of psychopathic disorders in American psychotherapist institute is updated and include two type for disorders axis I and II. SCID-I seven diagnostic groups of disorders of axis I include tempera psychopathic disorders, psychoneurotic, dependence on narcotic substance, anxiety and psychical disorders; eating disorder and adaptability have been assessed. Studying psychometric features of this tool show that its stabilization for deeply disorder better than weaker disorders and its credit are in range of 81 to 84 percent. Anyway this interview due to universality and exact adaptation with criteria of cognitive and statistical booklet of psychopathic disorders in American psychotherapist institute is more valid than other clinical criterion and it is one diagnostic and broad assessment standard about researching, legal and clinical subjects that are highly used (Sadock and Sadock, 2005). In Iran, Sharifi (2003) studied its stabilization and applicability for Iranian population. Findings show that cognitive adaptation for most diagnosis is medium to good (Capay more than 0/6) and most interviewers assessed applicability of Persian type of this tool. Bakhtiyari (Bakhtiari, 1999) use this tool for Iranian population and coefficient stability of retest after a week for SCID-I have been reported 95 percent (Mazaheri et al., 2007).

## 2.3 Questionnaire of Defensive Methods

This questionnaire evaluates defensive behavior by empirical assessment of derivative conscious defensive mechanisms in daily life (Sen Martini et al., 2004). This questionnaire have made according to hierarchal defenses model. For the first time this questionnaire codified by Band and his Colleague for studying defensive mechanisms in normal and patient people in 1983 that are include 88 materials and studied 24 mechanisms (Andrews et al.,1989; Muris & Merckelbach, 1996; Hayashi et al., 2004).

Band and his Colleague by analytical methods specified four defensive methods at the defensive mechanisms level such as:

1) Unadoptable method;
2) Deformation of mental image method;
3) Self sacrificed method;
4) Adaptable method (Sen Martini et al., 2004). After that they codifying this tool, they are studying relation between defensive methods of four groups of psychopathic disorders and also studied a group of normal people. Results of studies about distinction studied groups from each other and also distinct normal people from patients according to defensive methods are not satisfying. So Andrews and his colleague (Andrews et al., 1989) according to hierarchical diagnostic assessment based on reviewed context of third edition of cognitive and statistical booklet of psychopathic disorders in American psychotherapist institute and mentioned definitions about defensive mechanisms have reviewed questionnaire of defensive mechanisms and present questionnaire of defensive mechanisms that are contained 72 questions in third developed level, neuroticism and undeveloped have been assessed and because of inequality weak related materials to factor of other new type which is contain 40 question by Andrews and his colleague codified in 1993, that is contain 40 question and 20 defensive mechanisms which has assessed at three level (Andrews et al., 1989; Sinha & Watson, 2004). Coefficient Cranach’s alpha of questions every developed and undeveloped methods and criteria neurotic Persian form in one scholarship sample for all testee were respectively 0.75, 0.73, 0.74 for boy scholar 0.74, 0.74, 0.72 and for girls scholar 0.75, 0.74, 0.74 that is considered as illustrator satisfied internal homology for Iranian form of questionnaires’ defensive mechanisms (Besharat, 2007b).

Statistical analysis for describe data used percent, mean, standard deviation and for analysis correlation between groups used k-2 and ANOVA. Data analyzed by SPSS20 and significant level is 0/005.

## 3. Results Interpretation

In studied sample which is contained 390 tested, samples are as follow that is include 59/24% women, and 89% is disruptive family disorder.

**Table 1:** Comparison frequency of tetraploid groups separately according to Gender, level of education and Job

Variable Index		Developmental Disorder		Emotional disorder		Disruptive behavior disorder		Chi-square	
		Frequencies	Percent	frequencies	percent	frequencies	percent	x2	P
Gender	father	56	43.08	62	47.69	41	31.53	201.08	0.708
	mother	74	56.92	68	52.31	89	68.47		
	DIP	47	36.16	53	40.36	41	31.53		
	DIP UP	29	22.30	23	17.69	36	27.69		
Education	MC	35	26.92	33	25.38	34	26.15	601.09	0.901
	MA	19	14.61	21	16.15	16	12.30		
Job state	clack	41	31.53	72	55.39	69	53.07	201.08	0.708
	unemployed	89	68.46	58	44.61	61	46.92		

According to Table (2) maximum and minimum average age respectively related to patient affected by Developmental family and Emotional family disorder and in tested people have meaningful difference.

**Table 2:** Comparison average age of sextet groups

Group	Age rang	M	SD	F	P
Developmental	43 – 21	34/34	5/19		
Emotional	42 – 20	30/31	3/92		
Disruptive behavior	40 – 19	31/98	4/12	6.58	0.000
Total	43 – 19	32/44	4/87		

For studying used defensive mechanism models and comparison defensive mechanism models of this group used ANOVA and accordance with equal numbers of testee in groups, for compare pair groups used HSD test and the same subgroup, their results are present in following table.

Results of ANOVA and HSD test show that Table (3):

**Table 3:** Results of ANOVA between defensive mechanism and methods in compared groups

Variable Defense		Developmental		Emotional	Disruptive behavior		total		
		M	SD	M	SD	M	SD	F	P
undeveloped	Rationalization	19	2.7	18	2.5	18.1	2.6	34.2	0.126
	Projection	19.1	2.7	16.1	1.2	15.1	1.4	33.3	0.231
	Denial	21.1	3.0	18.1	2.6	19.1	2.9	36.8	0.123
	Omnipotence	10.4	1.8	9.1	2.3	13.6	1.9	34.2	0.098
	Devaluation	14.4	2.4	12.9	2.3	11.2	1.9	34.2	0.098
	Gozar	14.2	2.2	15.9	2.6	14.5	2.1	28.2	0.089
	Bodybuilding	25.2	3.7	19.2	2.7	18.2	2.9	35.3	0.000
	Autistic Fantasy	14.8	2.38	19.8	2.5	15.6	2.3	28.6	0.108
	Laye	14.9	2.8	13.5	2.4	18.5	2.4	34.9	0.146
	Passive aggression	19.1	2.7	16.7	1.2	15.1	1.4	33.3	0.321
	Displacement	13.7	2.6	12.7	1.6	13.4	2.9	11.1	0.356
	Compartmentalization	14.2	2.2	13.4	2.7	13.2	1.3	19.6	0.013
	TOTAL	39.1	3.8	23.1	3.6	19.4	3.5	25.9	0.079
Developed	Valayesh	5.4	2.1	3.7	2.7	6.4	2.3	11.2	0.018
	Sublimation	6.6	2.2	7.8	3.1	7.6	2.9	14.8	0.021
	Humor	4.3	1.5	6.5	2.3	4.9	1.7	29.2	0.192
	Anticipation	5.3	1.9	6.4	2.3	5.7	1.9	18.8	0.063
	TOTAL	6.6	1.9	7.4	1.5	7.7	2.4	21.2	0.068
Neurotic	false friendship	14.1	2.2	15.6	2.6	13.1	3.1	22.3	0.575
	Reaction formation	11.1	1.8	12.2	1.9	11.3	1.8	17.0	0.000
	Metallization	14.5	2.3	12.7	2.6	12.7	2.9	16.6	0.087
	Undoing	14.6	1.5	9.2	1.7	11.6	2.4	14.8	0.042
	TOTAL	12.6	2.3	11.1	3.7	9.8	3.5	18.8	0.937

Family of developmental disorder use more undeveloped defensive mechanism (39.1) that neuroticism patient (12.6) and developed methods (6.6) and maximum average in undeveloped defensive mechanisms include: Bodybuilding (25.2), Denial (21.1), and in defensive mechanism of psychoneurotic including from Undoing (14.6), Metallization (14.5) and false friendship (14.1) proportionally used more.

Family of emotional disorder so use more undeveloped defensive mechanism (23.1). Maximum average in undeveloped defensive mechanisms include: Denial (18.1), Autistic Fantasy(16.8) and Passive aggression(16.7) and in defensive mechanism of psychoneurotic including from friendship (15.6), Metallization (12.7) proportionally used more.

Family of Disruptive behavior disorder so use more undeveloped defensive mechanism (19.4). Maximum and minimum average in undeveloped defensive mechanisms include: Denial (19.2) and Devaluation (11.2). and in defensive mechanism of psychoneurotic including from friendship (13.1) and Reaction formation(11.3).

## 4. Discussion and Conclusion

According to researches of Cramer (Cramer, 2000) psychopathic disorders family with special defensive methods have been undeveloped and unadoptable and in nonclinical population defensive mechanism is infinitely more developed. Although defensive mechanism are normal and all use it, but if extremely used, it is caused obsessive behavior and repeated behavior and neuroticism, because for create and keeping defensive mechanisms we need more energy, as we are more defensive we need less mental power for satisfying action of character. of course, this is actually is its duty that create defensive mechanism, because it must avoid direct relation to instinctive request and defend himself against anxiety (Freud, 1905/1960; Fest and Fest, 2002) defensive mechanisms are distorting of reality and level of distortion in undeveloped defenses and neuroticism are more than developed defenses and level of cognitive distortion have reverse relation with consciousness as whatever level of cognitive distortion of defense become more proportionally consciousness, its conscious decrease and less effort for confront with cognitive distortion have been done(Haward, 2006; Freud, 1905/1953a). Though all defensive mechanisms are defend from him against anxiety, whereas everyone has defensive behavior, so these mechanisms are general and every defensive mechanism mixed with repression and everyone can cause psychopathic damage. Nonetheless, usually some mechanism for human is harmful and for society is harmless such as defensive mechanisms of neuroticism and some other mechanism is a barrier for understands reality and foreclose reasonable defensive of him like undeveloped defensive mechanism and sublimation defensive mechanism usually yield an interests for person and society. According to this description, in this research based on defensive mechanism in

Developmental, Emotional (Internalization), and Disruptive behavior (Externalization) disorders specified that:

Undeveloped defensive mechanisms in parent affected by Family of developmental disorder are the most used mechanism and in parent affected by Disruptive behavior (Externalization) disorders by average (19/04) is the least mechanism than other disease.
